# Phytochemical profile and nematicidal potential of essential oil from Algerian wild *Origanum vulgare* subsp. *glandulosum* Defs

**DOI:** 10.55730/1300-0152.2723

**Published:** 2025-01-27

**Authors:** Amina MEZERKET, Juan Emilio PALOMARES-RIUS, Souad BOUASLA, Henia SAIB

**Affiliations:** 1 Department of Natural Sciences LESN, Higher Normal School of Kouba (ENS) Cheikh Mohamed El Bachir El Ibrahimi, Algeries, Algeria; 2 Institute for Sustainable Agriculture (IAS), Higher Council for Scientific Research (CSIC), Cordoba, Spain; 3 Faculty of Sciences, University of Ferhat Abas, Campus El Bez, Setif, Algeria

**Keywords:** Wild grown oregano, oil composition, nematodes toxicology

## Abstract

**Background/aim:**

The root gall nematode *Meloidogyne incognita* constitute the most damaging species that infects many crops in Algeria. The intense use of harmful agricultural chemical products has incited research to develop alternative methods with natural and ecological advantages like essential oils extracted from plants. The objective of this study was to evaluate the efficacy of *Origanum vulgare* subsp. *glandulosum* Desf. (Lamiaceae) essential oil on the development of the root-knot nematode *M. incognita* in potted tomatoes.

**Materials and methods:**

In pot trials, we assessed the activity of *O. vulgare* subsp. *glandulosum* essential oil at two concentrations of 0.75 and 0.37 mg/L against *M. incognita*. These dilutions were applied in two treatments to soil: the preventive treatment (pretomato planting), and the curative treatment (posttomato planting), using an artificially inoculated tomato under controlled conditions.

**Results:**

The application of *O. vulgare* subsp. *glandulosum* essential oil was very effective at the pretomato planting treatment compared to the chemical treatments, and the inoculated control. We noted a reduction in number of roots and soil juveniles, galling index, and an increase in the tomato root and stem weights. The phytochemical screening of *O. vulgare* subsp. *glandulosum* revealed the presence of five classes of bioactive compounds (glycosides, saponins, flavonoids, tannins, and gallic tannins).

**Conclusion:**

This study showed a potential nematicidal effect of *O. vulgare* subsp. *glandulosum* essential oil on root-knot nematode.

## 1. Introduction

The tomato (*Solanum lycopersicum* L., Solanaceae) stands out as the most widely consumed vegetable fruit globally, attributed to its high nutritional content, including significant levels of vitamin C, minerals, and trace elements, with lycopene being a prominent antioxidant that has garnered considerable interest. It can be cultivated under diverse conditions, suitable for both fresh consumption and industrial processing (Naika et al., 2005). These practices have resulted in the emergence of various pests, notably root-knot nematodes (*Meloidogyne* sp.), which are considered one of the most harmful groups of parasites affecting plants worldwide. The primary symptom of the disease is the formation of tumors and knots on tomato roots (Rawal, 2020). Farmers predominantly rely on chemical products for soil disinfestation. However, agricultural chemicals present significant challenges, comprising the potential to poison plants, consumers, and groundwater (Zhang et al., 2018) and in many countries their use has been banned or seriously restricted. Nonetheless, to mitigate the reliance on synthetic pesticides, research has increasingly focused on plant-derived molecules and essential oils, which are gaining attention due to their ecological benefits (Isman, 2020). First developed by Arabs in the Middle Ages, approximately 3000 essential oils are currently recognized and used worldwide, with 300 specifically marketed in the pharmaceutical, agricultural, food, medical, fragrance, and cosmetics sectors (Bakkali et al., 2008). Additionally, they can affect a broad spectrum of species including insects (Giuliano et al., 2024), fungi (Allagui et al, 2024), bacteria (Truong and Mudgil, 2023), parasitic plants and weeds (Marcio et al., 2023). These essential oils comprise a complex mixture of various compounds, some of which exhibit nematicidal properties, including alkaloids, phenols, sesquiterpenes, diterpenes, and polyacetylenes (Catani et al., 2023; De Sousa et al., 2023). A considerable number of these chemical compounds have been identified and shown to inhibit or eradicate nematodes (Renco et al., 2014). Many authors indicated the nematicidal properties of garlic essential oil against *Pratylenchus sp*., *Globodera sp*., and *Heterodera schachtii* (Auger and Thibout, 2005). Moreover, *thymus vulgaris*, *Rosmarinus officinalis*, and *lavandula spp*. (Lamiaceae) essential oils showed a higher ovicidal and larvicidal potential on *Meloidogyne chitwoodi* (Kasapoğlu Uludamar, 2023). The grown wild *Origanum vulgare* subsp. *glandulosum* Desf. (Lamiaceae) is a plant commonly found throughout the Mediterranean region, particularly in the Algerian-Tunisian tell. It thrives in various areas of Algeria in its natural state. This endemic species is used in traditional Algerian as culinary herb. Moreover, it is considered as medicinal plant for treating severe diseases (Adouani and Boulaacheb, 2022).

Therefore, the present study aims to achieve the following objectives: i) To conduct a phytochemical screening of *O. vulgare* subsp. *glandulosum*, which is widely widespread in Algeria; and ii) To assess the toxic impact of this essential oil in preplanting and post planting treatment on tomato root gall nematodes.

## 2. Materials and methods

### 2.1. Nematode inoculum

The root-knot nematode used in our experiment was *Meloidogyne incognita*, a major pest in tomato. It was extracted from infested tomato roots collected in the Bab ezzouar region, located approximately 15 km east of Algiers, Algeria. Identification of the species was conducted based on perineal and morphological characteristics. The multiplication of the pure culture of *M. incognita* involved several stages: i) Preparation of tomato seedlings: the seeds employed were obtained from the tomato cv. Marmande, recognized for its susceptibility to the *Meloidogyne* genus. Seedlings with 4 to 6 leaves were subsequently transplanted into plastic pots with a diameter of 8 cm, and filled with sterilized soil. These pots were maintained in a greenhouse at Ecole Nationale Supérieure Agronomique (ENSA) Algiers, Algeria. experimental station, and kept at 24 ± 3 °C for about three months. ii) Acquisition of second-stage juvenile (J2) as described by the method of Hooper et al. (2005): J2s were obtained from egg masses collected from galls on the sampled tomato roots. The egg masses were placed in hatchers containing sterile distilled water for 2 to 3 days to facilitate juvenile hatching. iii) Inoculation: It involved the introduction of 2500 juveniles of *M. Incognita* (J2s of 48 h) at the base of each tomato seedling. This procedure was performed regularly to ensure a continuous supply of nematode egg masses, and juveniles throughout the duration of the experiment.

### 2.2. Plant material

The plant chosen for our experiment is the wild grown oregano, scientifically referred to as *O. vulgare* subsp. *glandulosum*, and belonging to the Lamiaceae family. This local species was collected during its flowering stage in the Bejaïa province (250 km north-east of Algiers, Algeria), and identified with voucher specimens of the herbarium of Botany Department, Ecole Nationale Supérieure Agronomique (ENSA), Algiers, Algeria. The origan leaves were dried for 7 to 10 days in the laboratory, where it is arranged in a thin layer, and open air out of the sunlight.

### 2.3. Essential oil extraction of *O. vulgare* subsp. *glandulosum*

The essential oil was obtained from the air dried plant by the hydrodistillation method (modified clevenger apparatus). This operation was performed at the laboratory of pharmaceutical group SAIDAL, located in Algiers, Algeria. This common technique is recognized for its effectiveness in extracting significant quantities of essential oil, as noted by Coppen (1995). The distillation process lasted for 3 h. We placed the dried plant material into a 2 L flask, which was filled to one-third of its capacity with water. The flask was heated using a balloon heater, generating steam that carried the essential oil components, which were condensed and cooled to a temperature range from 17 °C to 22 °C in a cooling apparatus before being collected in a recipe container. Following the recovery of the distillate, we proceeded to the decantation stage. This stage involved the use of a separating funnel, into which diethyl ether was introduced as a solvent. The mixture was then shaken, degassed, and allowed to settle until two distinct layers, water and oil were formed. The oil layer was filtered through anhydrous sodium sulfate. The tube was left exposed to air for a period from 24 to 48 h to facilitate the evaporation of the solvent. The essential oil was subsequently stored in a hermetically sealed dark glass bottle at a temperature of −4 °C for future use. The essential oil derived from *O. glandulosum* was used as a standard solution. It was diluted in 10% ethanol to achieve two concentrations of 0.75 and 0.37 mg/L, which were employed in our experiment.

### 2.4. In vivo, nematicidal effect of *O. vulgare* subsp. *glandulosum* essential oil

The experiment was conducted using 1.5 kg plastic pots with a diameter of 15 cm. The test setup included two distinct treatments: i) Treatment before planting, ii) Treatment after planting. The pots were filled with a sterilized mixture of soil and potting compost (2:1 v/v). Three-week-old tomato plants were transplanted at a density of one plant per pot for the postplanting treatment, alongside two chemical treatments: fenamiphos and ethoprophos, applied at doses of 0.05 g of nematicide containing 10% of fenamifos/ plant and 0.09 g of nematicide containing 10% of fenamifos/plant (based on standard concentrations 30 kg/ha and 50 kg/ha, respectively, recommended from the supplier). For the preplanting treatment, every pot was previously infested with 2500 juveniles (J2s of 48 h) in a deep hole in the middle of the soil. Two days following the inoculation, every pot was treated with 10 mL of each dose of oregano essential oil (0.75 and 0.37 mg/L) placed in the same hole, and subsequently covered with plastic film for a duration of 3 days prior transplanting. Concerning the postplanting treatment, the application of the oregano essential oil doses and chemical treatments was realized few days after inoculation of the tomato seedlings in a deep hole surrounding the plant stem. A nematode-inoculated control was also performed. Five replicates were applied for each treatment; every replicate comprised a single potted plant. The experiment was repeated twice. These pots were arranged in a completely randomized block design within a greenhouse conditions 25 ± 2 °C. They were regularly irrigated and fertilized with NPK. The following variables were assessed after a period of two months: the number of juveniles and eggs per root (Coolen et Herd, 1972), the number of juveniles in soil per plant (Dalmasso, 1966), the reproduction factor (RF = final population/initial population), the weight of fresh aerial tomato plant, the weight of fresh tomato roots, and the root galling index (GI) was evaluated from 1 to 10 according to the scale developed by Bridge and Page (1980).

### 2.5. Phytochemical screening analysis of *O. vulgare* subsp. *glandulosum*

Phytochemical analyses were performed on two distinct occasions to confirm the composition of our essential oil. The first evaluation was carried out at the SAIDAL laboratory, and this was followed by a second analysis at the Ethnobotany and Natural Substances Laboratory (LESN) located within the Ecole Normale Supérieure (ENS) Kouba, Algiers, Algeria. These analyses involved testing either the powdered plant material to identify the presence of quinones, alkaloids, glucosides, and coumarins, or using an infused solution to detect leucoanthocyanins, anthocyanins, tannins, flavonoids, and saponins, in accordance with the protocol established by Bruneton (1999). For the infusion preparation, 20 g of the plant powder was mixed with 100 mL of distilled water and boiled for 15 min. The resulting filtrate was then diluted to a final volume of 100 mL with distilled water.

#### 2.5.1. Test for anthocyanins

We added 10 drops of hydrochloric acid to 5 mL of the plant extract. The reaction produced a red colour in the presence of anthocyanins.

#### 2.5.2. Test for leuco-antocyanins

We added 2 g of plant powder to 20 mL of a propanol/hydrochloric acid mixture (1/1). These compounds were heated in a boiling water bath for a few minutes. A red colour developed in the presence of the leuco-anthocyanins.

#### 2.5.3. Test for tannins

About 10 drops of a ferric chloride solution were combined with 5 mL of the plant extract. The reaction produces a blue-black colour in the presence of the tannins.

##### 2.5.3.1. Test for catechetic tannins

We mixed about 15 mL of the infusion with Stiasny’s reagent. The reaction gave a red colour in the presence of catechetic tannins.

##### 2.5.3.2. Test for gallic tannins

A few drops of ferric chloride were combined with the filtrate of plant extract saturated with sodium acetate. The reaction gave a dark blue colour in the presence of the gallic tannins.

#### 2.5.4. Test for flavonoids

We added 5 mL of hydrochloric acid, and a magnesium ribbon to 5 mL of the plant extract. The reaction turned into orange-red in the presence of flavonoids.

#### 2.5.5. Test for quinones

##### 2.5.5.1. Test for free quinones

About 2 g of plant powder was moistened with 2 mL of hydrochloric acid and chloroform. The reaction became red in the presence of free quinones.

##### 2.5.5.2. Test for combined quinones

The resultant filtrate of 2 g of plant powder was mixed with 5 mL of sulfuric acid 2N, and chloroform. The formation of red colour indicated the presence of combined quinones.

#### 2.5.6. Test for saponins

Around 2 mL of the plant extract was mixed with a few drops of lead acetate. The appearance of a white precipitate revealed the presence of saponins.

#### 2.5.7. Test for alkaloids

About 5 g of plant powder was macerated with 20 mL ammonia (1/2) in 50 mL ether chloroform (3/1) for 24 h. A red precipitate determined the presence of alkaloids.

#### 2.5.8. Test for glucosides

A few drops of sulphuric acid were added to 2 g of plant powder. The formation of a brown-red coloration revealed the presence of glucosides.

#### 2.5.9. Test for coumarins

Approximately 10 drops of the plant extract were combined with 20 mL of ethyl alcohol. A few drops of potassium hydroxide (10%), and hydrochloric acid (10%) were added to the mixture. The apparition of cloudiness indicates the presence of coumarins.

### 2.6. Statistical analysis

Data analysis was conducted using ANOVA with EXELSTAT v10. Differences in means among the treatments, as well as between the treatments and control groups, were assessed using Turkey test Statistical significance was established at p < 0.05.

## 3. Results

### 3.1. Phytochemical screening test of *O. vulgare* subsp. *glandulosum*

The phytochemical analysis of *O.vulgare* subsp. *glandulosum* identified five distinct groups of bioactive secondary metabolites.The main several primary compounds were tannins, gallic tannins, saponins, and glucosides, which were found in substantial quantities. Conversely, flavonoids were detected only in trace amounts within this species. Furthermore, the analysis revealed a complete absence of catachetic tanins, anthocyanins, leuco-anthocyanins, alkaloids, senosides, and coumarins, as detailed in Table 1.

### 3.2. In vivo, nematicidal activity of *O. vulgare* subsp. *glandulosum* essential oil

The results of the effect of Origano essential oil on *M. incognita* and plant growth are shown in Table 2. All treatments revealed a nematicidal effect against *M. incognita* compared to inoculated control. The highest numbers of nematode juveniles in soil and roots were recorded in the inoculated control. However, we found significant differences among treatments in the two temporal points of applications. The application of *O. vulgare* subsp. *glandulosum* essential oil before planting, mainly at concentrations of 0.75 mg/L, proved to be highly effective across all majority assessed parameters (gall idex, fresh shoot weight, fresh root weight, numbers J2s in soil/plant, numbers of juveniles and eggs/root and reproduction factor). The reduction in eggs and juveniles of *M. incognita* in roots was observed to be more than 75% from the inoculated control. This reduction was notably less pronounced in the treatments applied after planting. Among the chemical treatments, fenamiphos exhibited greater efficacy compared to ethoprophos. Nevertheless, the preplanting application of *O. vulgare* subsp. *glandulosum* oils at a concentration of 0.37 mg/L yielded results comparable to those of fenamiphos. The ethoprophos, on the other hand, displayed effects similar to those of the postplanting treatment at the same concentration. Additionally, the highest gall index was noted in the inoculated control, averaging 7. The RF in preplanting treatment at 0.75 mg/L was found to be the lowest compared to the inoculated control (a reduction of 75% in RF), which reached a rate of 21.82. A significant difference was observed among the various treatments when compared to the infested control. The impact of *O. vulgare* subsp. *glandulosum* essential oil on the growth of tomato plants was also evaluated. Regarding fresh root weights, the roots treated with fenamiphos show a higher weight than those that received preplanting treatment. In contrast, the fresh weight of roots treated with ethoprophos is higher when compared to the preplanting treatment at a concentration of 0.37 mg/L. Finally, the infested control group exhibited the lowest weight. The findings indicated that the fresh stem weights of tomato plants were again significantly greater in the fenamiphos followed by the preplanting treatment at 0.75 mg/L.

## 4. Discussion

The results indicated that the preplanting treatment significantly influenced the population of *M. incognita* when compared to the control group. The application of *O. vulgare* subsp. *glandulosum* essential oil reduced considerably the levels of nematode parasitism. This effectiveness can be attributed primarily to the pretreatment of juveniles with the essential oil tested, in conjunction with the use of a plastic cover (preventive mode), which allows the juveniles to remain exposed to volatile plant compounds for an extended period (Valdes et al., 2011). Furthermore, the bioactive mixtures found in oregano leaves have demonstrated a nematicidal effect against *M. incognita*. The use of a tannin solution (phenols) reduced the nematode egg hatch and has repellent-nematostatic (or disorientation) effect on juveniles applied during the planting stage or prior to transplanting that may disrupt the capacity of nematodes to locate root systems and possibly diminishing the damage inflicted on the plants (Maistrello et al., 2010). In fact, approximately ten different compounds were found in essential oils, including diterpenes, fatty acids, tannins, glycosinolates, isothiocyanates, phenols, sesquiterpenes, and thienyls (Teixeira et al., 2019). These main elements are known for their toxic properties on nematodes in numerous studies (Kundu et al, 2021; Catani et al., 2023). Many authors reported that phenols such as flavonoids and simple phenols, and monoterpenoids such as thymol possess larvicidal and ovicidal effects against several species of *Meloidogyne* (Li et al., 2013; Faria et al., 2015). Our chemical screening of *O. vulgare* subsp. *glandulosum* oil showed an important diversity in the chemical composition (glycosides, saponins, flavonoids, tannins, and gallic tannins), as indicated in Table 1. This diversity of compounds could be attributed to multiple factors and could be variable depending on variety, chemotype, plant origin, harvesting time, plant parts utilized, and the extraction method employed for the essential oil (Moghaddam and Mehdizadeh, 2017). Additionally, climatic conditions and the intensity of plant metabolic processes may also affect the chemical composition of the oils (Laftouhi, et al., 2023). In this way, Wuyts et al. (2006) indicated that flavonoids and simple phenolic compounds, including phenylpropanoids, and monoterpenoids like thymol resulted in complete mortality of *M. incognita* and prevent hatching. These substances also function by inhibiting hatching and decreasing the populations of *Radopholus similis* and *M. incognita* larvae, exhibiting an LD50 of 46 μg/mL following 72 h of nematode exposure. The same authors emphasize that phenylpropanoids are crucial in the defense and resistance mechanisms of plants against diseases and pests, particularly nematodes. Moreover, saponins extracted from the tissues of five *Medicago* species have demonstrated significant in vitro efficacy against many nematodes such us *Xiphinema index*, *M. incognita*, and *Globodera rostochiensis*. In experiments involving potted tomato plants, the application of saponins resulted in a decrease in both root and soil population densities of *M. incognita* when compared to untreated controls, specifically at rates of 20 and 40 g/kg of soil. This treatment also led to an overall enhancement in plant growth and yield performance (D’Addabbo et al., 2020). Following Chin et al. (2018), flavonoids exert various influences on nematode populations, such as either attractants or repellents, inhibit the hatching of eggs, and may even lead to the death of these organisms. Additionally, the incorporation of *Brassica macrocarpa* leaves, recognized for their elevated glucosinolate content, into the soil around tomato roots affected by *Meloidogyne* populations at a concentration of 300–650 mol/m^2^ led to a significant decrease in gall formation by as much as 50% (Argento et al., 2019). As stated by Augereau (2008), the level of interest in an extract correlates directly with its activity level. Consequently, to identify active components, it is essential to decompose the extract into simpler fractions, guided by the physicochemical characteristics of its constituent molecules. In the present study, treatment with the *O. vulgare* subsp. *glandulosum* essential oil regularly reduced the reproduction rate of *M. incognita* compared to inoculated control. The data for our chemical screening of *O. vulgare* subsp. *glandulosum* essential oil agree with results of numerous studies conducted in Algeria. In fact, the chemical composition of the same species from Bejaia with gas chromatography mass spectrometry (GC-MS) analyses revealed its richness of phenolic monoterpenes, with carvacrol content of 63.7% and thymol content of 36.7% (Chikhoune, 2004). As indicated by the same author in a similar investigation on *O. vulgare* subsp. *glandulosum* from Sétif (east of Algiers, and south of Bejaia, Algeria), thymol and carvacrol were identified as the primary constituents, with respective concentrations of 38.8% and 32.9%. Furthermore, a total of 18 compounds were detected in this species, with p-cymene and y-terpinene being the most significant at 7.9% and 5.1%, respectively. In agreement with literature findings, another study of the phytochemical analysis of *O. vulgare* subsp*. glandulosum* from the Sétif region showed a significant concentration of monoterpenes like carvacrol (47%), g-terpine (13.4%), p-cymene (11.2%), and thymol (6.6%) (Belhattab et al., 2004). Several researches have exposed the high antimicrobial activity of essential oils from various species of oregano which was attributed to their richness in phenolic compounds, specifically carvacrol and thymol (Chun et al., 2005). According to Ntalli, (2010), these two main compounds found in oregano particularly decreased the infection of *M. incognita*. As evidenced by studies conducted both in vitro and in vivo, the presence of these terpenes (carvacrol, thymol, and σ-cymene) typically enhanced the nematicidal properties of an essential oil (Catani, 2023). Referring to Nasiou and Giannakou (2023), the application of thymol resulted in over 90% mortality of second-stage juveniles of *M. javanica* (J2s) after 96 h of exposure at a concentration of 500 μL/L, as well as a 59.7% inhibition of nematode hatching. In addition, the use of thymol in pots at low concentrations reduced the eggs, and female numbers at 150 and 300 μL/L on tomato roots. Moreover, the essential oil of oregano was evaluated for its fungicidal, bactericidal, antiviral, and antioxidant properties (Leyva-López et al., 2017). An experiment demonstrated that the interaction action of carvacrol and thymol with a tyramine receptor; a neuroactive compound in nematodes such as SER-2, can trigger a series of signaling events that ultimately lead to the death of nematodes (Lei et al., 2010). Similarly, a neurotoxic mode of action has been highlighted specifically for both fenamiophos, and ethoprophos that functioned as inhibitors of acetylcholinesterase. However, fenamiphos exhibited systemic action, showing enhanced mobility and solubility in soil. In contrast, ethoprophos acted through contact and is recognized for its high reversibility among organophosphorus nematicides (Hartwig et al., 2001).

## 5. Conclusion

The study findings indicated that the essential oil derived from *O. vulgare* subsp. *glandulosum* exhibited a significant nematicidal activity against *M. incognita*. The application of essential oil before planting serves as a more effective preventive strategy against *M. incognita* compared to after planting. Nevertheless, additional in-depth research is required to isolate the active components of this plant, and understand its mechanism of action for the potential development of biopesticide. Finally, it is desirable to conduct many tests using various medicinal plants from different families as nematicidal agents.

## Figures and Tables

**Table 1:** Phytochemical analysis revealing various compounds existing in screened plant.

Chemical components	*Origanum vulgare* subsp. *glandulosum* Desf.
Leucoanthocyanins	a−
Anthocyanins	−
Catachetic tanins	−
Galic tanins	d + + +
Tanins	+ + +
Free quinones	−
Combined quinones	−
Sapononins	+ + +
Senosides	−
Alkaloids	−
Flavonoids	c + +
Glucosides	+ + +
Coumarins	−

Each level (indicator) was based on colour intensity of each test reaction that indicates the presence or the absence of the compounds. The results can be read qualitatively using the following level classification:

a −: Absence of compounds.

b +: Moderately rich in compounds.

c ++: More or less rich in compounds.

d +++: Very rich in compounds.

**Table 2:** Effect of the pre and postplanting treatments with oregano essential oil on the tomato progression factors and on reproduction of *M. incognita* under greenhouse conditions.

Treatments and doses	Gall index (1–10)	Fresh shoot weight (g)	Fresh root weight (g)	Number J2s in soil/plant	Number of juveniles and eggs/root	Reproduction Factor^b^ (Pf/Pi)
**Preplanting**						

**0.37 mg/L**	3.4 bc	45.00 c^a^	20.20 c	934.80 b	14,766.20 b	6.28 b
**0.75 mg/L**	2.8a	56.00 de	23.20 e	650.40 a	13,224.00a	5.55 a

**Postplanting**						

**0.37 mg/L**	4.6d	32.00 b	19.00 b	1263.60 cd	27,432.20 d	11.48 d
**0.75 mg/L**	3.8bc	40.00 c	20.00 c	1125.60 c	17,736.00 c	7.54 c
**Fenamiphos (0.05 g/plant)** c	3.0b	62.00 e	23.80e	608.40 a	16,193.52 bc	6.72 bc
**Ethoprophos (0.09 g/plant)** c	4.0c	52.40 d	22.40 d	1678.80 d	33,496.96 d	14.07 d
**Inoculated control**	7.0e	22.60 a	18.60 a	2961.60 e	51,596.40 e	21.82 e

a Each value represents the mean of five replicate plants per treatment using the combination of two different experiments. Means followed by the same letter are not significantly different at p <0.05 according to Turkey’s test within each treatment including inoculated control.

b Reproduction factor: PF (final population including the number of nematodes from roots and soil/initial population (2500 inoculated J2s)).

c g of commercial product with 10% active ingredient each.
